# Medio-Lateral and Flexion-Extension Gap Imbalances in Mechanically Aligned Total Knee Arthroplasty Using Measured Resection Technique in Korean Patients: 3D Simulation

**DOI:** 10.3390/jcm10040845

**Published:** 2021-02-18

**Authors:** Byung Woo Cho, Ji Hoon Nam, Yong Gon Koh, Kwan Kyu Park, Kyoung Tak Kang

**Affiliations:** 1 Department of Orthopedic Surgery, Severance Hospital, Yonsei University College of Medicine, 50-1 Yonsei-ro, Seodaemun-gu, Seoul 03722, Korea; chobw0704@yuhs.ac; 2 Department of Mechanical Engineering, Yonsei University, 50 Yonsei-ro, Seodaemun-gu, Seoul 03722, Korea; namjh8901@naver.com; 3 Joint Reconstruction Center, Department of Orthopaedic Surgery, Yonsei Sarang Hospital, 10 Hyoryeong-ro, Seocho-gu, Seoul 06698, Korea; osygkoh@gmail.com

**Keywords:** Korean patients, imbalance, morphometry, total knee replacement

## Abstract

Background: It is well known that the measured resection (MR) technique in mechanically aligned (MA) total knee arthroplasty (TKA) generates significant gap imbalances, but little is known about whether this applies to the knees of Asian patients. The aim of this study was to evaluate the medio-lateral and flexion-extension gap imbalances and to find the most optimal posterior femoral condyle resection method for operating on the knees of Asian patients. Methods: In total, 738 magnetic resonance imaging (MRI) scans of consecutive patients who underwent TKA were obtained. Four posterior femoral condylar resection methods were used: alignment by the surgical transepicondylar axis (TEA), Whiteside’s line (WSL), 3° external rotation to the posterior condylar axis (PCA), and flexion-extension axis (FEA). Results: For the medial compartments, there were significant differences between the flexion and extension gaps in the varus knee group in all four methods, but there were no differences between the flexion and extension gaps in the valgus knee group. For the lateral compartment, all the methods showed significant differences except for WSL of the valgus knee group and FEA of the varus knee group. Conclusions: In Asian patients, the use of the MA MR technique inevitably leads to medio-lateral or flexion-extension imbalances. Therefore, surgeons should consider which methods can minimize imbalances and choose the best method within the technically possible range.

## 1. Introduction

### 1.1. Background

Mechanically aligned (MA) total knee arthroplasty (TKA) allows the distal femur and the proximal tibia to be resected perpendicular to the mechanical axis of each bone to achieve neutral alignment [1]. In this process, the anatomical diversity of each patient is ignored and new kinematics are formed. This has been indicated as one of the causes of dissatisfaction after TKA [2,3]. Nonetheless, it is the most widely practiced technique as it has the advantages of high reproducibility and ease of use.

The rotational alignment of the femoral components in TKA not only affects the occurrence of patellar tracking and anterior notching but also the symmetry of the flexion gap [4,5]. The measured resection (MR) technique is used to determine the external rotation in MA TKA, and the rotation degree is determined through the bony landmarks [6,7]. The landmarks mainly referenced in the MR technique are Whiteside’s line (WSL) [5], the surgical transepicondylar axis (TEA) [8], and 3° external rotation to the posterior condylar axis (PCA) [9]; however, it is unclear which of these landmarks is superior for use in the MR technique [10].

### 1.2. Rationale

In a three-dimensional (3D) simulation study using 1000 computed tomography (CT) images, Blakeney et al. reported that the MA MR TKA frequently generated many imbalanced gaps in both extension and flexion, and that flexion gap imbalances were more frequent in TEA than in PCA [11]. In a 3D simulation study using 50 CT images, Gu et al. reported that in MA MR TKA a ≥ 2 mm instability in a compartment between 0° of extension and 90° of flexion that was uncorrectable by collateral ligament release with PCA was less frequent compared to WSL and TEA [12]. These two studies mainly pointed to the limitations of the MA technique. However, their results also imply that the PCA method has the lowest gap-imbalance rate among the external rotation methods they observed. Of course, it is difficult to determine the superiority only from these results because the gaps were evaluated simply by resected bone thickness. However, it may be helpful in finding a method that minimizes ligament release to correct imbalances. Since the previous studies were conducted on Western populations, it remains unknown whether their results can be applied to knees in the Asian population. In general, the knees of Asians are generally smaller in size than those of Caucasians, and the knee’s 3D morphology is reportedly different [13,14,15,16]. Therefore, when evaluating the gap imbalances of Asian patients, there may be differences from the results of studies conducted on Western populations.

The first aim of this study was to evaluate the medio-lateral and flexion-extension gap imbalances when MR MA TKA was performed in the knees of Asian patients using 3D simulation analysis. To more realistically reflect the cartilage status of osteoarthritic patients, magnetic resonance imaging (MRI) scans were used to make 3D models, unlike in previous studies. Secondly, when resecting the posterior femoral condyle, we tried to determine the most optimal method amongst those available (WSL, TEA, PCA, and flexion-extension axis (FEA)) for operating on the knees of Asian patients.

## 2. Patients and Methods

Institutional review board approval was obtained for this study. Seven hundred ninety-two consecutive patients who were undergoing TKA at our institution were given an MRI scan. All the subjects had osteoarthritis (OA) of Kellgren–Lawrence grades 3 and 4. The exclusion criteria included any history of osteotomy or fracture to the affected limb. As a result, 738 patients, comprising 631 varus and 107 valgus patients, were included in the final analysis.

Subjects’ characteristics such as age, sex, hip–knee–ankle angle (HKA), and body mass index (BMI, kg/m^2^) were recorded and are listed in Table 1. MRIs were acquired using an MRI scanner 1.5-T (Achieva 1.5 T; Philips Healthcare, Best, Netherlands). They were obtained using a high-resolution slice with a thickness of 1 mm in the sagittal plane of the tibiofemoral knee joint and a slice with a thickness of 5 mm in the axial plane for the hip and ankle joints. Under non-fat saturation conditions, the MRIs consisted of axial proton density sequences. A high-resolution setting was used for the spectral pre-saturation inversion recovery sequence (echo time, 25.0 ms; repetition time, 3590.8 ms; acquisition matrix, 512 × 512 pixels; number of excitations (NEX), 2.0; field of view, 140 × 140 mm). This MRI method used in patient-specific instruments allowed us to effectively obtain a 3D reconstructed model [17]. The MRI images were imported into a 3D-modeling software (Mimics version 17.0; Materialize, Leuven, Belgium) and segmented to construct 3D bone and cartilage models of the femur and tibia. The 3D reconstruction reproducibility analysis was performed similarly to that in our previous study [18].

**Figure 1:**
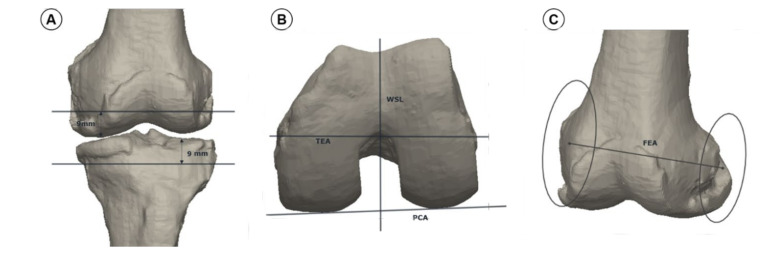
Method of surgical simulation with 3-dimensional model. (**A**) Bone resection methods. The resection depth was 9 mm from most distal and posterior condyle for the femur and most proximal plateau for the tibia; (**B**) Definition of Whiteside’s line (WSL), surgical transepicondylar axis (TEA), and posterior condylar axis (PCA); (**C**) Definition of flexion-extension axis (FEA).

The mechanical axes of the femur and tibia were created in the 3D reconstructed model. The mechanical axis of femur was defined as a line between hip center and intercondylar notch. The mechanical axis of tibia was defined as a line between the center of proximal tibia and the center of ankle. HKA was defined as the angle between the femoral and tibial mechanical axis, wherein 0° was defined as neutral, more than 0° was defined as varus, and less than 0° was defined as valgus. The distal resection was conducted using two distal planes that were perpendicular to each femoral and tibial mechanical axis. The resection depth was 9 mm from the most distal condyle for the femur and the most proximal plateau for the tibia (Figure 1A). Four posterior femoral reference methods were used (Figure 1B,C): alignment by surgical TEA, WSL, 3° of external rotation to the PCA, and FEA. TEA was defined as a line connecting the most prominent lateral epicondylar projection and medial epicondylar sulcus [19]. WSL was defined as a line connecting the deepest point on the trochlear groove and intercondylar notch [20]. PCA was defined as a line tangent to the most prominent point or posterior condyle [20]. The FEA was defined as a line connecting the center of the medial and lateral best-fitted circle on the sagittal plane [21]. Posterior cutting was performed using planes perpendicular to the femoral or tibial distal planes and parallel to each posterior femoral reference axis. The posterior resection depth was 9 mm from the most posterior condyle.

The gap distances were calculated as the sum of resected bone thicknesses, namely, the extension-medial, extension-lateral, 90° flexion-medial, and 90° flexion-lateral gap distances. Using these gap distances, four imbalances were calculated as described previously in literature [11]: medio-lateral imbalance (MLI, lateral gap minus medial gap) in extension, MLI in 90° flexion, flexion-extension imbalance (FEI, extension gap minus flexion gap) in the medial compartment, and FEI in the lateral compartment. Two independent observers subsequently measured the flexion and extension gaps on the MRI. The observers were blinded to the subjects’ medical histories. To evaluate intra-observer and inter-observer variabilities, 3D MRI scans were remeasured > 1 week after the initial measurements by the first and second observers. The intra-observer and inter-observer errors were 0.90 and 0.93, respectively, as calculated using the intraclass correlation method.

Statistical analysis was performed using R (version 3.6.3, R Foundation for Statistical Computing, Vienna, Austria) to calculate the arithmetic means, standard deviation, and significance probability. A two-sample *t*-test was used to compare means between two independent groups. An analysis of variance (ANOVA) and post hoc analysis were used to compare three or more means. The Chi-squared test was used to compare the ratio in categorical data. The significant differences were regarded when the *p*-value was less than 0.05. Power analysis was conducted using G power 3.1 (Heinrich Heine Universität Düsseldorf, DE). The input parameters were the medial and lateral flexion gaps of a varus knee. The alpha value was 0.05, and calculated power was 100%. In comparison, previous studies had used a target power of 80% [22,23].

## 3. Results

### 3.1. Medio-Lateral Imbalance in Extension

**Figure 2:**
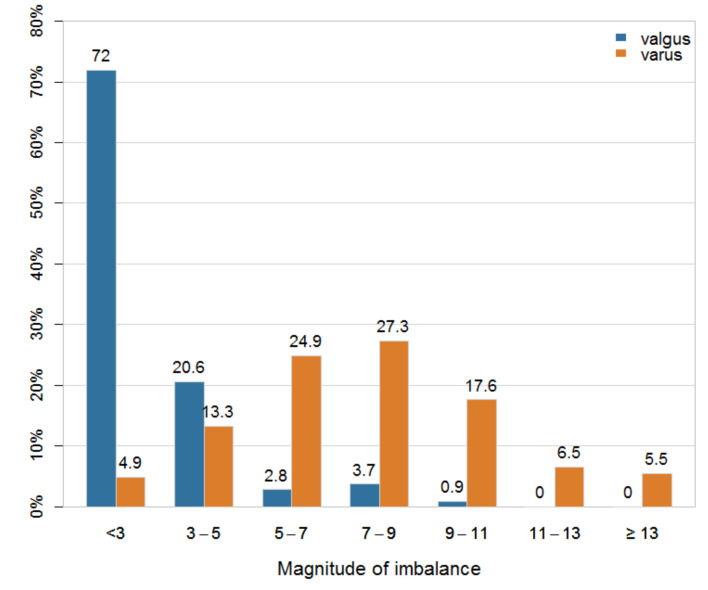
Medio-lateral imbalances in extension gaps according to the varus/valgus knee group.

The average MLI in extension was 7.7 ± 3.1 mm in the varus knee group, which was significantly greater than the average 0.5 ± 3.3 mm observed in the valgus knee group (*p* < 0.001, Table 2). In the varus knee group, 89.5% had an MLI of 3 mm or greater, compared to 28% in the valgus knee group. As the magnitude of the imbalance increased, the corresponding proportion of patients decreased in the valgus knee group, while in the varus knee group, there was an initial increase followed by a decrease (Figure 2).

### 3.2. Medio-Lateral Imbalance in Flexion

**Figure 3:**
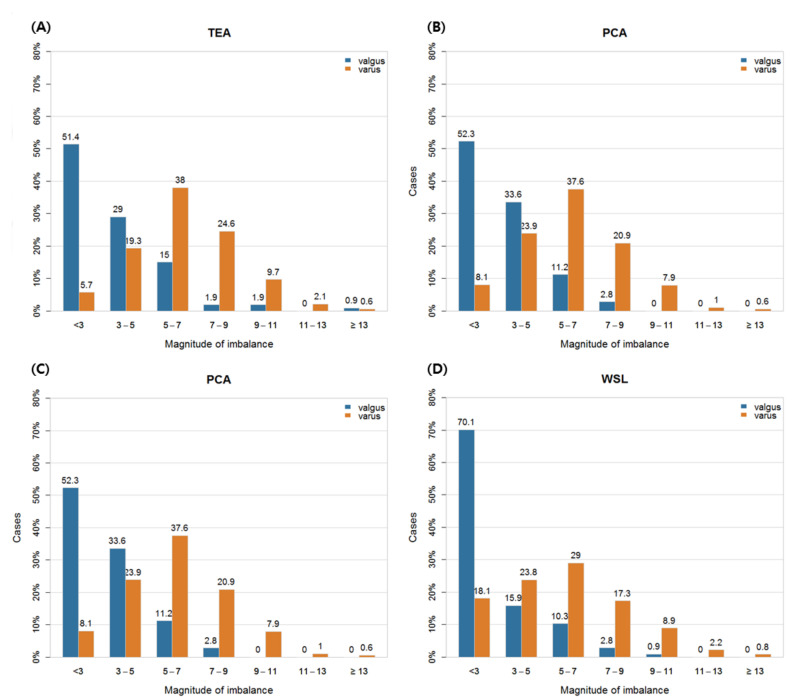
Medio-lateral imbalances (MLI) in flexion gaps according to the varus/valgus knee group. Distribution of MLI based on (**A**) surgical transepicondylar axis (TEA), (**B**) Whiteside’s line (WSL), (**C**) posterior condylar axis (PCA) and (**D**) flexion-extension axis (FEA).

The flexion gaps according to TEA, PCA, WSL, and FEA are shown in Table 3. In all four methods, the MLIs in the varus knee group were significantly greater than those in the valgus knee group (*p* < 0.001, respectively). The post hoc results for the average MLI in the varus knee group showed that there was no difference between TEA and PCA, the WSL gave a lower average MLI, and FEA produced the largest variance. In the valgus knee group, the post hoc results for the average MLI showed no difference between TEA, PCA, and FEA, while WSL gave a significantly lower result. In other words, the MLIs at flexion for both varus and valgus knee groups were minimal in WSL. The proportion of patients for each magnitude of imbalance in each method is shown in Figure 3. In all four methods, as the magnitude of the imbalance increased, the corresponding proportion of patients decreased in the valgus knee group, while in the varus knee group, there was an initial increase followed by a decrease.

### 3.3. Flexion-Extension Imbalance

**Figure 4:**
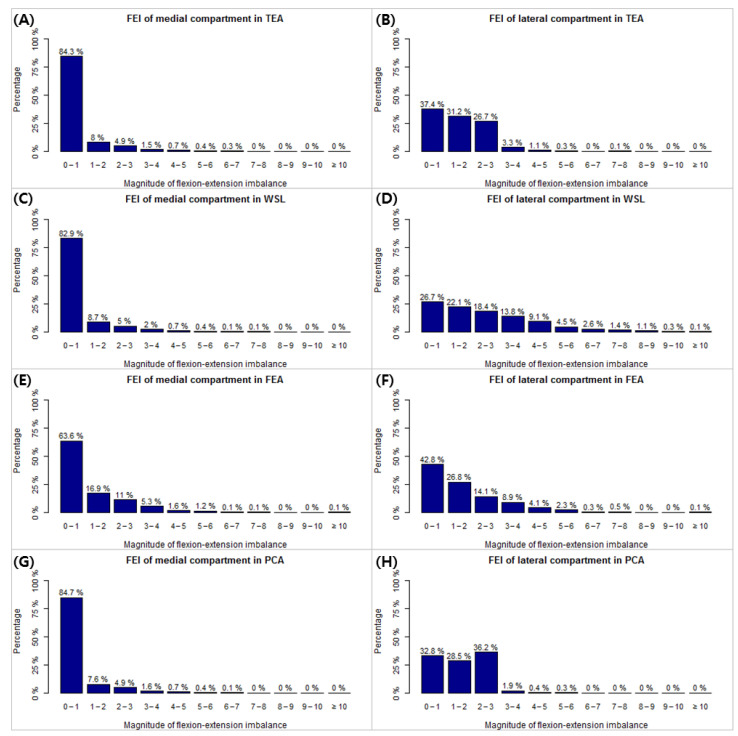
Flexion-extension imbalances (FEI) according to the varus/valgus knee group and the four posterior femoral condylar resection methods. FEI distribution of (**A**) medial compartment based on surgical transepicondylar axis (TEA), (**B**) lateral compartment based on TEA, (**C**) medial compartment based on Whiteside’s line (WSL), (**D**) lateral compartment based on WSL, (**E**) medial compartment based on flexion-extension axis (FEA), (**F**) lateral compartment based on FEA, (**G**) medial compartment based on posterior condylar axis (PCA) and (**H**) lateral compartment based on PCA.

The FEIs for each method are shown in Table 4. For the medial compartments, there were significant differences between the flexion and extension gaps in the varus knee group in all four methods, but there were no differences between the flexion and extension gaps in the valgus knee group. For the lateral compartment, all the methods had significant differences except for WSL of the valgus knee group and FEA of the varus knee group. The distribution of FEI according to the four methods is shown in Figure 4.

The proportion of patients with medial and lateral compartment flexion-extension gap differences simultaneously showing no imbalance is shown in Table 5. In the varus knee group, TEA and PCA showed the highest balance rates when the reference was set to 3 mm (95.1% and 96%, respectively), and TEA showed the highest balance rate when the reference was set to 2 mm and 1 mm (66.7% and 35.3%, respectively). In the valgus knee group, PCA had the highest balance rate in all references.

## 4. Discussion

This study shows that a significant and variable imbalance inevitably occurs when using MA MR TKA in the knees of Asian patients. For extension gaps, many imbalances that require a release to create a rectangular gap occur only by resection according to MA, coinciding with the results of previous studies [11]. WSL was found to be the most advantageous for minimizing MLI in flexion, but either TEA or PCA was found to be more effective for minimizing FEI; hence, it is impossible to choose the optimal method to satisfy all balances at once. In other words, if the MA MR technique is used, imbalances inevitably occur, resulting in soft tissue strain and ligament release in some cases. This may be one of the causes of dissatisfaction occurring in the 10–20% of cases after TKA [24,25,26,27].

Prior to the interpretation of our data, the characteristics of this study to be considered were as follows. First, since our study was analyzed through modeling using MRI, the cartilage thickness was reflected in the results. Since CT images cannot display the cartilage, the results are different from those of real OA patients. If both the medial femoral condyle and the medial tibial condyle have cartilage defects and the lateral compartment is intact, the effect of a cartilage thickness of total 4 mm [28] in the lateral compartment gets ignored in the CT scan. Therefore, the lateral gap is underestimated compared to the actual gap, leading to the MLI (lateral gap minus medial gap) values being reduced by about 4 mm, and the PCA and FEA internally rotate more than the actual angles. Second, this study was conducted on the knees of Asian patients. Asian knees show many anthropometric differences compared to Caucasian knees. It is known that Asians have a larger angle between TEA and PCA than Caucasians [13], and the medial proximal tibial angle (MPTA) is also larger [29]. Since our study performed bone resection based on the maximum thickness, medial tibial bone resection was reduced in knees with large MPTA. These two characteristics showed differences from the results of previous literature using CT.

In the CT study of Blakeney et al., the number of patients with flexion or extension MLI was the highest in the 0–3 mm interval in the varus and valgus knee group [11]. In this study, the valgus knee group showed similar results, but the varus knee group showed the largest number of patients at around 7 mm (Figure 2, 3). As aforementioned, using MRI produces larger lateral gaps than those of CT studies owing to the thickness of the cartilage, and the larger MPTA in the knees of Asians makes smaller medial gaps than those of Caucasians owing to less medial tibial resection. For these reasons, MLI in the varus knee group in this study is generally larger than that reported by Blakeney et al.

Gu et al. described that the proportion of ≥ 2 mm FEI was the lowest in PCA compared to TEA or WSL, in a CT simulation study of Caucasians [12]. However, this result also does not consider the thickness of the cartilage; hence, the lateral gap was underestimated. Moreover, because the study of Gu et al. set the standard of bone resection as a minimum of 6 mm, it seems that the proportion of imbalance is different from that of this study, in which the maximum thickness was set to 9 mm.

There is no consensus regarding the permissible extent of MLI and FEI in TKA. As for MLI, our study defined the rectangular gap as the medio-lateral balance, but there is a growing recognition that a slight asymmetric gap (both in extension as well as in flexion) is more desirable than a rigorous symmetry because it resembles the kinematic of the normal knee [30]. Even if the ideal degree of asymmetry is revealed in the future, our comprehensive data can be used accordingly. As for FEI, some studies recommend that the flexion-extension gap asymmetry be within 2 mm [31,32], but one other study sets the outlier to 3 mm [33]. Thus, we analyzed the proportion of patients whose medial and lateral compartment flexion-extension gap differences simultaneously showed no imbalance by references of ≤ 1, ≤ 2, and ≤ 3 mm (Table 5). In the varus knee group, TEA had the highest balance rate at ≤ 2 mm and was similar to PCA at ≤ 3 mm. In the valgus knee group, PCA had the highest balance rate at all references. MLI can be easily corrected with additional techniques, such as soft tissue release, but FEI cannot. Therefore, choosing the method with the highest balance rate in the FEI rather than the MLI would lead to a better outcome. However, in these cases, it may be necessary to release ligaments to correct inevitably generated MLI.

In TKA, gaps are determined by the degree of bone resection and soft tissue laxity. Since the effect of soft tissue was not considered when defining the gaps in this study, the measured gaps might be underestimated from the actual gaps. However, Bellemans et al. reported that soft tissue disturbance in the extended knee does not occur when the preoperative deformity is less than 10° [34], and McAuliffe et al. reported that almost no real disturbance occurs in the coronal plane soft tissue envelope when flexing at 90° on OA knees with varus deformity less than 15° [35]. Since the patient group and the study design are different, it is impossible to set the exact numeric reference; however, taken together, the effect of soft tissue can be considered negligible in the knees without severe deformity. Therefore, the results of this study may be applicable to most OA knees.

This study has several limitations. First, only Korean patients were examined. Future investigations should include other Asia–Pacific populations to establish a general database in this region. Second, this study does not provide postoperative clinical outcomes as it did not study patients who had undergone TKA. Third, the MRI images used to construct the 3D representation of the distal femur and proximal tibia could have led to inevitable errors in the computation model. Using MRI did, however, allow reconstruction of soft tissues (e.g., articular cartilage), and the accuracy in 3D reconstruction could be improved by following a protocol described in a previous study [36].

Nevertheless, this study had many strengths, including employing a large dataset of 738 cases, reflecting the actual state of the cartilage using MRI, and comparing four different posterior condylar resection methods. Moreover, despite the limitation of the MA MR technique, this study suggests the method that could lead to the best outcome.

## 5. Conclusions

In conclusion, in the knees of Asian patients, the use of the MA MR technique inevitably leads to medio-lateral or flexion-extension imbalances. Therefore, surgeons should consider which methods can minimize imbalances and choose the best method within the possible range.

## Figures and Tables

**Table 1:** Comparison of the demographics according to the varus and valgus knee groups.

Parameter	All Patients (*n* = 738)	Varus (*n* = 631)	Valgus (*n* = 107)	*p*-Value
	Mean ± SD (Range)	Mean ± SD (Range)	Mean ± SD (Range)	
Age	70.4 ± 7.2 (0, 87)	70.2 ± 7.3 (0, 87)	71.7 ± 6.0 (59, 81)	0.12
BMI (kg/m^2^)	23.1 ± 3.5	23.1 ± 3.4	22.8 ± 3.9	0.53
Male/female	133/605	109/522	24/83	0.20
Hip–knee–ankle angle	6.4 ± 5.5 (−15.7, 22.7)	8.2 ± 3.6 (0.1, 22.7)	−3.9 ± 3.4 (−15.7, 0)	NA

SD, standard deviation; BMI, Body mass index; NA, not applicable; male/female row indicates counts.

**Table 2:** Medio-lateral imbalances in extension gap according to the varus and valgus knee groups.

		Varus	Valgus	*p*-Value
		Mean ± SD (Range)	Mean ± SD (Range)	
Extension Gap	Imbalance (Lat – Med)	7.7 ± 3.1 (−9.9, 18.2)	0.5 ± 3.3 (−10.1, 1.09)	*p* < 0.001
	Med	9.8 ± 1.8 (2.7, 18)	13.1 ± 2.2 (8.6, 18)	
	Lat	17.5 ± 1.8 (8.1, 25.6)	14 ± 1.7 (7.9, 19.5)	

Med, medial gap; Lat, lateral gap; SD, standard deviation.

**Table 3:** Medio-lateral imbalances in 90° flexion gap according to the varus and valgus knee groups and posterior femoral condylar resection methods.

	Flexion Gap	Varus	Valgus	*p*-Value
		Mean ± SD (Range)	Mean ± SD (Range)	
TEA	Imbalance (Lat–Med)	6.4 ± 2.3 (−6.4, 14.7)	2.6 ± 3.7 (−4.4, 14.5)	*p* < 0.001
	Med	10.2 ± 1.5 (9, 18)	13.3 ± 2.6 (3.5, 18)	
	Lat	16.6 ± 1.4 (9.8, 23.7)	15.9 ± 1.1 (13.6, 18)	
PCA	Imbalance (Lat–Med)	6.0 ± 2.3 (−7.2, 14.3)	2.0 ± 3.1 (−7.2, 8.8)	*p* < 0.001
	Med	10.2 ± 1.5 (8.2, 18)	13.4 ± 2.4 (9, 18)	
	Lat	16.2 ± 1.2 (9.8, 23.3)	15.4 ± 1.3 (8.8, 17.8)	
WSL	Imbalance (Lat–Med)	5.5 ± 3.1 (−12, 15.2)	1.2 ± 3.3 (−7.7, 9.8)	*p* < 0.001
	Med	10.2 ± 1.6 (4.8, 18)	13.1 ± 2.3 (9, 18)	
	Lat	15.7 ± 2.3 (6, 24.2)	14.3 ± 2.4 (8.6, 18.8)	
FEA	Imbalance (Lat–Med)	8.0 ± 3.3 (−6, 20.8)	3.6 ± 5.1 (−11.2, 12.1)	*p* < 0.001
	Med	9.5 ± 2 (−2.8, 18)	12.8 ± 3.1 (7, 18)	
	Lat	17.6 ± 1.9 (8, 26.3)	16.4 ± 2.5 (6.8, 20.4)	

TEA, surgical transepicondylar axis; PCA, posterior condylar axis; WSL, Whiteside’s line; FEA, flexion-extension axis; Med, medial gap; Lat, lateral gap; SD, standard deviation. Post hoc results of delta comparison in varus: WSL < PCA = TEA < FEA. Post hoc results of delta comparison in valgus: WSL < PCA = TEA = FEA (PCA < FEA).

**Table 4:** Flexion-extension imbalances according to the posterior femoral condylar resection methods in the medial and lateral compartments.

	Medial Gap				Lateral Gap			
	Extension	Flexion 90°	Delta (Ext–Flex)	*p*-Value	Extension	Flexion 90°	Delta (Ext–Flex)	*p*-Value
TEA								
Varus	9.8 ± 1.8 (2.7, 18)	10.2 ± 1.5 (9, 18)	−0.4 ± 0.9 (−6.3, 1.9)	<0.001	17.5 ± 1.8 (8.1, 25.6)	16.6 ± 1.4 (9.8, 23.7)	0.8 ± 1.5 (−5, 3.4)	<0.001
Valgus	13.1 ± 2.2 (8.6, 18)	13.3 ± 3 (3.5, 18)	−0.2 ± 1 (-4.1, 6.8)	0.52	14.2 ± 1.9 (7.9, 19.5)	15.7 ± 1.5 (8.8, 18.7)	−1.5 ± 1.7 (−7, 2.6)	<0.001
PCA								
Varus	9.8 ± 1.8 (2.7, 18)	10.2 ± 1.5 (8.2, 18)	−0.4 ± 0.9 (−6.3, 3)	<0.001	17.5 ± 1.8 (8.1, 25.6)	16.2 ± 1.2 (9.8, 23.3)	1.2 ± 1.3 (−3.8, 3.2)	<0.001
Valgus	13.1 ± 2.2 (8.6, 18)	13.4 ± 2.4 (9, 18)	−0.3 ± 0.7 (−4.1, 0.4)	0.42	14.2 ± 1.9 (7.9, 19.5)	15.4 ± 1.3 (8.8, 17.8)	−1.2 ± 1.6 (−5.6, 2.6)	<0.001
WSL								
Varus	9.8 ± 1.8 (2.7, 18)	10.2 ± 1.6 (4.8,18)	−0.3 ± 1.1 (−6.3, 7.5)	<0.001	17.5 ± 1.8 (8.1, 25.6)	15.6 ± 2.3 (6, 24.2)	1.8 ± 2.6 (−4.9, 10.7)	<0.001
Valgus	13.1 ± 2.2 (8.6, 18)	13.1 ± 2.3 (9, 18)	0 ± 0.9 (−4.8, 3.4)	0.93	14.2 ± 1.9 (7.9, 19.5)	14.3 ± 2.4 (8.6, 18.8)	−0.1 ± 2.4 (−6.9, 8.2)	0.67
FEA								
Varus	9.8 ± 1.8 (2.7, 18)	9.5 ± 2 (−2.8,18)	0.3 ± 1.6 (−6, 14)	0.005	17.5 ± 1.8 (8.1, 25.6)	17.6 ± 1.9 (8, 26.3)	−0.1 ± 1.9 (−5.8, 15.7)	0.26
Valgus	13.1 ± 2.2 (8.6, 18)	12.8 ± 3.1 (7, 18)	0.3 ± 1.9 (−5.2, 4.7)	0.40	14.2 ± 1.9 (7.9, 19.5)	16.4 ± 2.5 (6.8, 20.4)	−2.2 ± 2.6 (−7.9, 5.7)	<0.001

TEA, surgical transepicondylar axis; PCA, posterior condylar axis; WSL, Whiteside’s line; FEA, flexion-extension axis.

**Table 5:** Proportion of patients with medial and lateral compartment flexion-extension gap differences simultaneously showing no imbalance.

	Medial and Lateral Side		
	Ext-Flex Imbalance ≤ 1	Ext-Flex Imbalance ≤ 2	Ext-Flex Imbalance ≤ 3
TEA			
Varus	35.3%	66.7%	95.1%
Valgus	29%	57%	77.6%
WSL			
Varus	22.7%	43.6%	63.2%
Valgus	27.1%	58.9%	77.6%
FEA			
Varus	30.6%	62.3%	83%
Valgus	13.1%	24.3%	43%
PCA			
Varus	30.7%	59.6%	96%
Valgus	33.6%	60.7%	86.9%

TEA, surgical transepicondylar axis; WSL, Whiteside’s line; FEA, flexion-extension axis; PCA, posterior condylar axis.

## Data Availability

The data presented in this study are available on request from the corresponding author. The data are not publicly available due to privacy.
